# What Endgame for the Deglobalisation Narrative?

**DOI:** 10.1007/s10272-022-1085-y

**Published:** 2022-12-16

**Authors:** Simon J. Evenett

**Affiliations:** MBA - Intern. Trade and Economic Developm., University of St. Gallen, Blumenbergplatz 9, 9000 St. Gallen, Switzerland

**Keywords:** F13, F23, F52

## Abstract

From the perspective of international economic governance, other than casting aspersions on the judgement of those that negotiated previous multilateral trade accords and the accession of China to the World Trade Organization, the deglobalisation narrative is silent on how to reform that organisation — or what to salvage from existing global trade rules.

In his highly regarded book of pandemics, published before COVID-19 was declared a global emergency, Yale Professor Frank Snowden (2019) argued that plagues and the like have unleashed profound social and economic forces that have significantly reordered societies. History may be repeating itself, at least in respect to some public sector and private sector decision-makers' attitudes towards globalisation.

Documenting shifts in opinion is not easy – and indeed may not matter that much until it translates into action. Nevertheless, the growing salience of a narrative of "deglobalisation" is difficult to ignore. In the four years before the pandemic, the *Factiva* database recorded on average 850 media mentions of the term deglobalisation.[1] Since 2020, deglobalisation has been mentioned on average 4,534 times per year – from 1 January 2022 to 20 November 2022, this term has been referred to 7,323 times in media outlets around the world. Something is afoot.

So what is the deglobalisation narrative? Does it have any grounding in fact? And, critically, what is its endgame? Given the dense web of cross-border commercial ties that knit together national economies, and the implications for our living standards, there is a lot at stake. The purpose of this article is to report on what the author has learned when investigating the emerging narrative of deglobalisation.

1 Searches were conducted for the American spelling of this term and for the translations of this term into Chinese, French, German, Japanese, Portuguese, Russian and Spanish.

* Comments on this piece are welcome – please send them to simon.evenett@unisg.ch.

**Simon J. Evenett**, University of St. Gallen; and St. Gallen Endowment for Prosperity Through Trade, Switzerland.

## Pinning down the deglobalisation narrative

One hurdle to understanding the deglobalisation narrative is that few have spelt it out in any length. Yet, a review of many media mentions of this term leaves the impression that this narrative has gained a life of its own – which could become problematic if the very narrative alone persuades policymakers to take damaging steps that turn deglobalisation into a self-fulfilling prophecy.

For sure, there have been critiques of global trade rules, of the consequences of trade reform and of international economic integration more generally, but they predate the emergence of the deglobalisation narrative. So what, if anything, is new here?

One useful place to start is with the writings of Rana Foroohar, Global Business Columnist and Associate Editor of the *Financial Times*. In addition to her weekly columns, a longer piece in that newspaper titled "My guide to a deglobalising world" (published on 21 October 2022), an article published in the November/December 2022 edition of *Foreign Affairs* (Foroohar, 2022a) and possibly in a forthcoming book (Foroohar, 2022b), Foroohar has gone further than most in spelling out what she sees as deglobalisation.

The central diagnosis appears to be:

> What is clear is that globalization is in retreat, at least in terms of trade and capital flows. The 2008-9 financial crisis, the pandemic, and the war in Ukraine all exposed the vulnerabilities of the system, from capital imbalances to supply chain disruptions to geopolitical turmoil. (Foroohar, 2022a, 141)

The central prediction appears to be:

> Countries now want more redundancy in their supply chains for crucial products such as microchips, energy, and rare earth minerals… All these shifts suggest that regionalization will soon replace globalization as the reigning economic order. Place has always mattered, but it will matter more in the future. (Foroohar, 2022a, 141)

The last point is at the core of Foroohar's critique of pre-pandemic thinking about globalisation: "Neoliberalism's agnosticism about place is striking", she writes (Foroohar,

2022a, 138). Supporters of pre-pandemic globalisation also erred in the following ways: "Counting on autocratic governments for crucial supplies was always a bad idea. Expecting countries with wildly different political economies to abide by a single trade regime was naïve" (Foroohar, 2022a, 145).

Another valuable point of reference in understanding the apparent shift in certain policymakers' thinking is Leonard (2021). Noting the resort to export bans at the onset of the COVID-19 pandemic, as well as prior attempts to limit the export of rare earth minerals and the use of bilateral trade measures to show disapproval of the foreign policy and other stances of foreign governments, Leonard argues that the instances of "weaponising" commercial ties have made officials realise that globalisation can be a source of risk as well as gain.

The imperative to manage such risks – in particular, reconfiguring supply chains so that production takes place at home or at least in allied countries – appears to have altered thinking about the relative benefits of cross-border sourcing. The sense that some officials in particular in Western governments were profoundly shocked by the resort to export curbs comes through clearly in Leonard's book.

## Is the deglobalisation narrative a reliable guide to policymakers and corporate executives?

It matters what the prevailing zeitgeist is as time-pressed leaders look for reliable shortcuts to facilitate decision-making. But how much of the deglobalisation narrative is grounded in fact? And, if it is, what decisions follow or, at least, what logic should govern those decisions?

### Retreat?

The notion that globalisation is in retreat has been carefully examined empirically. This matter was investigated in a sequence of four blog posts by Richard Baldwin in August and September 2022. Baldwin (2022a) concludes that the narrative that globalisation has peaked and is going into reverse is "overly simplistic". He argues: "In short, the 'globalisation has peaked' storyline is lazy, but there is a highly energetic reality behind it. The globalisation of markets for goods is no longer rising as it had been between the 1990s and the mid-2000s" (Baldwin, 2022a).

With respect to services trade, Baldwin (2022d) is emphatic: it is increasing and for good reasons. He argues:

> The divergence between the growth of services versus goods happened because digital technology opened the door to trade in intermediate services, and high-income countries have few or no barriers to this sort of exports. India, for instance, performed its service-export miracle without signing a single trade agreement. (Baldwin, 2022d)

As for the unwinding of supply chains, Baldwin (2022c) shows that the picture here is mixed. Industrialised countries are as engaged as ever in exporting intermediate goods, while their propensity to source them from abroad has been falling since the Global Financial Crisis. Baldwin (2022c) also shows that the complexity of supply chains – both domestic and international – has diminished over time, as measured by the share of manufacturing value added in gross production. These findings show that private firms can and do adjust without the need of government fiat.

The finding that globalisation is not in broad retreat does not imply that cross-border commercial ties are growing at a fast pace. The World Trade Monitor[2] publishes monthly data on goods import volumes, which is a good proxy for internationally contestable market access (for goods). During the years 2011-19, this measure of market access grew 1.5%-2% per year in industrialised economies and around 4% per annum in emerging markets. This represents a significant slowdown compared to the go-go years before the Global Financial Crisis, where the comparable percentages were 4.5%-5% and 12%, respectively. It is not for nothing that before the COVID-19 pandemic hit, *The Economist* (2019) magazine christened this phase of globalisation "slowbalisation".

There is also evidence of faltering foreign direct investment (FDI) inflows. When new flows of FDI are benchmarked against other sensibly chosen flows – namely, world GDP, world investment levels or world trade – the former has not kept up with the expansion of the latter three (Evenett and Fritz, 2021). This trend is discernible since the Global Financial Crisis, although inevitably there is year-to-year fluctuation.

Furthermore, outside of the Middle East, by 2015 the average returns on FDI by US multinationals in every other emerging market region had converged to levels earned by US subsidiaries operating in the European Union. This finding implies there is no longer a premium for exposure to greater policy and regulatory risks attendant in lower per-capita income nations. If this pattern applies to other nations' multinationals, then it may account for the shift in corporate investment towards projects at home rather than abroad (Evenett and Fritz, 2021). It is noteworthy

2 The regular reports of the World Trade Monitor can be obtained at https://www.cpb.nl/en/world-trade-monitor-july-2022.

that the evidence on the falling premia on returns to FDI in emerging markets predates the COVID-19 pandemic and applies in regions not associated with geopolitical rivalry with the United States.

What are the right conclusions to draw from this data? First, there are many different types of cross-border commercial ties and some are doing better than others (contrast cross-border e-commerce with FDI). This cautions against generalisation about the state of globalisation. Second, the retrenchment seen in industrial country sourcing of inputs and the shift of corporate investment towards home markets started before the COVID-19 pandemic and the emergence of the deglobalisation narrative.

Third, to the extent that this retrenchment reflects shifting risk assessments by corporate executives since the Global Financial Crisis, it pours cold water on any suggestion that businesspeople were oblivious to the risks – including political risks – arising from international commerce.

## Vulnerabilities exposed?

Central to demands to reconfigure supply chains is that current international sourcing practices left exposed nations vulnerable to shortages induced by foreign export restrictions and other measures to weaponise trade.

The onset of the COVID-19 pandemic resulted in a surge in demand for certain medical consumables and equipment. According to the Essential Goods Monitoring Initiative,[3] by mid-April 2020 a total of 71 customs territories had imposed some type of export control on medical goods. However, by 1 July 2020 that total had fallen to 52 jurisdictions, suggesting that many governments concluded that these curbs were ineffective or, worse, counterproductive.

The notion that imposing export curbs was inevitable is belied by the fact that Australia, Canada and Japan somehow managed to tackle the coronavirus without banning local manufacturers from fulfilling export orders. That democracies as well as autocracies banned exports of medical goods does not sit well with the deglobalisation narrative either. Nor does the fact that regional allies (in particular within Europe) curbed exports as well.

Another awkward fact for the deglobalisation narrative is that more governments turned to global markets to meet their medical goods needs once the COVID-19 pandemic hit their societies (see Figure 1). Dozens of governments took

Figure 1

**Export curbs on medical goods mushroomed at the start of the COVID-19 pandemic, but more steps were taken to ease sourcing from abroad**

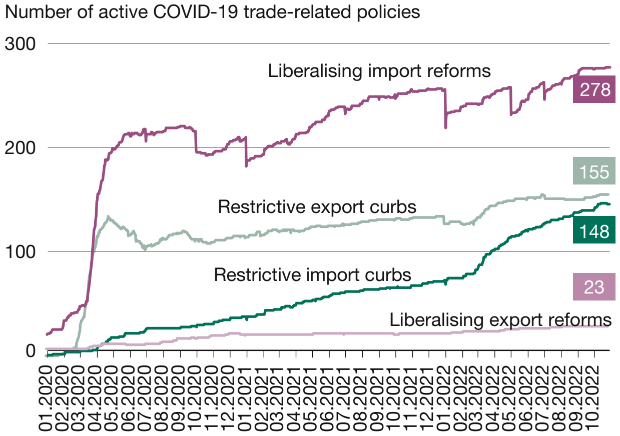

Source: Global Trade Alert, November 2022 release.

steps to ease imports of needed medical items. Over 200 such import reforms were in force by 8 May 2020 that had been implemented since the start of 2020. The total number of reforms to import policy that remained in force – tariff cuts, relaxation of import quotas, etc. – continued to rise throughout 2020. In short, trade was seen as part of the solution to shortages of medical goods – which is hard to square with talk of trade-related vulnerabilities.

Subsequent research also revealed just how few medical goods Western nations sourced primarily from China (Evenett, 2020; Guinea and Forsthuber, 2020) and just how diversified sourcing patterns of "essential medical goods" were in practice.[4]

Seen from the perspective of late 2022, however, it is the potential weaponisation of food exports that has contemporary salience. Russia's five month-long blockade of shipments of Ukrainian grain through the Black Sea is a case in point. Given that previous spikes of global food prices resulted in riots and other forms of political instability in certain net food-importing developing countries, any weaponisation of food trade is a serious matter. So what does the evidence on trade policy actions towards food, agri-food products and fertiliser reveal?

3 To be clear, the author is involved in the execution of this initiative as well as the Global Trade Alert mentioned later.

4 For evidence from the European Union, see Guinea and Forsthuber (2020).

Again, for evidence I turn to the findings of the Essential Goods Monitoring Initiative, upon which Figure 2 was constructed. There have been a significant number of trade policy steps taken this year, in particular since the invasion of Ukraine on 24 February 2022. As of this writing, 156 measures that restrict or ban exports of food are in force. However, many were implemented before the invasion of Ukraine.

In fact, since the invasion of Ukraine, 171 measures to restrict, discourage or ban food exports have been implemented worldwide. The Russian Federation is responsible for 44 of these steps, only six of which were formal export bans.[5] Russia's allies are responsible for another four measures. Other large autocracies are responsible for another nine restrictive export measures. As far as Russia's foes are concerned, Ukraine has implemented four export restrictions covering food products so far this year. The sanctions packages imposed by Western nations and Japan that implicate food trade must be added to this list.

What is also evident from Figure 2 is the even larger number of reforms of import policies towards food, agri-food and fertiliser products currently in force. A total of 210 steps to ease the importation of food have been taken worldwide since the invasion of Ukraine. These steps were taken by 58 customs territories, including three multi-country customs unions. Russia, its allies and other autocracies undertook 33 of these 210 import reforms.

Examination of the list of nations responsible for these import reforms reveals that it would be difficult to make any generalisations about which political systems tend to be responsible for easing food trade. Perhaps what matters more is that, similar to the case of medical supplies, governments of all stripes have taken steps that facilitate sourcing from world markets. This is very hard to square with a narrative of reducing vulnerabilities to foreign sourcing – although it must be conceded that the growing number of import restrictions on food and fertilisers (also shown in Figure 2) is not inconsistent with this part of the deglobalisation narrative.

Those advancing the deglobalisation narrative often refer to perils of import dependence on "crucial products" (as Foroohar did) or "critical materials". What evidence is there about sourcing patterns of these goods by liberal market economies? One useful source of evidence is the annual publication by the United States Government

Figure 2

**Governments have not relied solely on export restrictions to address food insecurity**

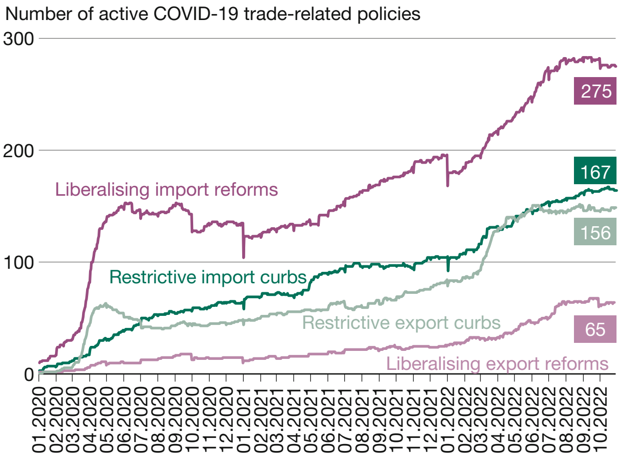

Source: Global Trade Alert, November 2022 release.

(USG, 2022) of Mineral Commodity Summaries. This publication focuses on the production and sourcing of minerals and commodities that are not fuel.

It is noteworthy that, in the 2022 edition, no mention is made of export restrictions in the section devoted to trade policy-related matters. In fact, the phrase "export ban" appears once in the document and, then, only in reference to a ban legislated by the United States on mercury in 2008. There are no references in the entire report to export quotas, nor to export taxes or to export restrictions imposed by foreign governments. The 25 references to shortages in the 2022 edition of this report are associated almost entirely with COVID-19 shutdown restrictions, extreme weather events and the lack of availability of container shipping – and not to trade policy or weaponising trade.

As for import dependence, the 2022 edition of the Mineral Commodity Summaries identified 50 minerals where the United States is both a net importer and where net imports account for half or more of US consumption (use) in 2021. The report observes:

> China, followed by Canada, supplied the largest number of these nonfuel mineral commodities. The countries that were the leading sources of imported mineral commodities with greater than 50% net import reliance were: China, 25 mineral commodities; Canada, 16 mineral commodities; Germany, 11 mineral commodities; South Africa, 10 mineral commodities; and Brazil and Mexico, 9 mineral commodities each. (USG, 2022, 5)

5 Russia frequently changes its export taxes on wheat, barley and corn in ways that few (if any) have linked to geopolitical considerations. Similarly, Argentina and Indonesia regularly change export taxes on a limited range of food products.

Only one country mentioned above is not a democracy. China is the largest import source in 18 of 25 cases where it is designated a "major import source". In each of the 25 of these cases, there is at least one US trading partner that is unquestionably a democracy which has also been listed as a major import source.

According to the USG (2022), Russia is a "major import source" for six minerals that the United States is reliant on to support half or more of its domestic use – but in each of these cases Russia is never the largest import source. In each case where Russia is listed as a major import source, there is at least one democratic trading partner of the United States that is listed as another major import source.

Furthermore, in this US government publication there is not a single mineral where net imports account for more than half of the total use by the United States with only autocracies listed as major import sources. In light of these findings, it is difficult to make an evidence-based case that autocracies have a strangle hold over the supply of key minerals – including rare earths – to the United States.

Overall, whether it is needed medical supplies, food, fertiliser or minerals, there are at best examples of occasional import dependencies on nations with different governance systems. Even when such dependencies exist, it does not imply that governments in exporting nations weaponise those trade links.

Even if governments attempt to weaponise trade, there are alternative suppliers that can expand production. Indeed, one of the more interesting empirical findings this year concerning global wheat supplies is that the reduction in supplies from Ukraine (part of which Russia was responsible for) was offset four times over by higher wheat exports from Argentina and Brazil (Glauber et al., 2022). Just because weaponisation is possible does not mean it must have far-reaching consequences.

## Naïve expectations about the multilateral trade regime?

The argument is frequently heard from Western and Japanese analysts and officials that China's World Trade Organization membership has not resulted in it adopting a market-based development model. Expectations that this would happen were naïve, we are now told. One implication is that only democratic, liberal market economies can be relied upon to comply with the spirit and the letter of the extant global trade rules. Those rules are based on the principle of non-discrimination, or equal treatment of domestic and foreign suppliers.

Figure 3

**The pot calls the kettle black: Liberal democracies violate the non-discrimination principles of the world trading system too**

Share of world goods trade covered by policies that favour local firms

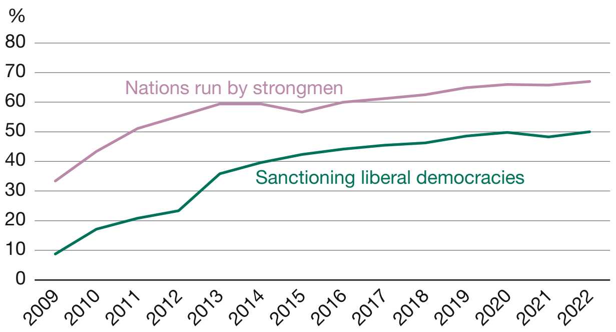

Note: The "Sanctioning liberal democracies" include the G-7 nations, the other members of the European Union, Australia, South Korea and New Zealand. The "Nations run by strongmen" are China, India, Russia and Turkey.

Source: Global Trade Alert, 2022.

One way to evaluate the implication mentioned in the previous paragraph is to examine shares of global goods trade covered by public policies that favour national firms (and likely violate the principle of non-discrimination) implemented by two groups of nations: the democracies that have sanctioned Russia this year[6] and the nations currently led by "strongmen" (in this case, China, India, Russia and Turkey) that are not typically aligned with liberal democracies. The goods trade coverage shares were computed using policy interventions recorded in the extensive database of unilateral policy intervention that may affect international commerce assembled by the Global Trade Alert team.[7]

Figure 3 reports the increases in global goods trade covered by policy interventions that favour local firms since the start of 2009. It does not make for comfortable reading. By now, half of global goods trade takes place between nations where one or more policy intervention by the sanctioning liberal democracies has tilted the commercial playing field in favour of local firms. This share has grown each year since the Global Financial Crisis and so predates the policy response to the COVID-19 pandemic. The policy interventions taken by the "strongmen" that favour local firms and exporters now implicate two-thirds of world goods trade. This share too has grown over time.

6 Taken here to include the G-7 nations, the other members of the European Union, Australia, South Korea and New Zealand.

7 More information about this independent monitoring initiative, including accounts of the methodology used, can be found at https://www.globaltradealert.org/about.

Fealty to multilateral trade principles is a matter of degree and is not determined solely by the nature of a jurisdiction's economic or political order.

In sum, when it comes to wishful thinking, there is no better place to start than the holier-than-thou attitude of certain analysts and officials in the liberal democracies concerning their national compliance with the non-discrimination principle of the world trading system. As the quaint expression goes, everyone has snow on their boots.

### Regionalisation replacing globalisation?

The potential for more intra-regional trade varies significantly across regions over the world economy. According to the United Nations Conference on Trade and Developmen, in 2020, 67.7% of European trade took place within that region. Intra-regional trade accounted for 58.3% of trade by Asian nations. For every other region, less than 30% of their trade was intra-regional (for the North American region the percentage stands at 29.3%).[8] In light of such statistics, proponents of the deglobalisation narrative essentially envisage far-reaching changes to international trade flows and reconfiguration of supply chains across the Atlantic and Pacific Oceans. Yet, it is worth bearing in mind that the United States is widely regarded as having relatively limited integration into world goods markets in the first place.

Having more local firms supplying local markets appears to be central to the deglobalisation narrative. Here it is worth recalling just how few firms actually engage in international trade in leading Western nations. In the United States, for example, 221,580 firms imported goods in 2020 (down 1.4% from the 2019 total) and 271,705 firms exported that year. A total of 189,607 firms both exported and imported that year. To put these numbers in perspective, the US government reports that over 5.5 million firms do not export.

In 2020, just under 60% of US firms that export do so to just one other nation. Another 24.4% of US exporters ship goods to between two and four nations. Firms that export to 50 or more nations account for just 0.4% of American exporters. The concentration of import sourcing is even higher among US importers.[9] When American firms engage in international trade, it is with counterparties in few other trading partners.

As for US multinationals, a total of 38,747 of their subsidiaries had net incomes above $25 million in 2019. Eight percent of those subsidiaries (3,101 in fact) were located in the strongmen nations mentioned above – including 1,951 in China and 631 in Russia. Less than 2.7% of the total value of assets invested by US multinationals in these 38,747 subsidiaries are located in the strongmen nations.[10]

In sum, the degree of US firm engagement in foreign markets is limited to start with. A wholesale retreat – or a retreat to North American markets – would not mean much for many American firms. Proponents of the deglobalisation narrative emphasise the "local": in reality, most American businesses have only been interested in the local. For other regions of the world economy, where the degree of integration into world markets is higher, retreat could have more far-reaching implications for corporate strategy.

## Concluding remarks

A narrative has taken hold on both sides of the Atlantic that contends that deglobalisation is happening or that it needs to happen. This narrative has the flimsiest foundation in fact. Remarkably, its proponents have been allowed to advance their arguments based on what they regard as telling examples, impressions gleaned at workshops and conferences of like-minded souls, surveys conducted by consulting companies that happen to sell advice to firms reconfiguring their supply chains, and by invoking fears that inevitably arise when the world is facing a sequence of crises.

The saying "A lie can travel halfway around the world before the truth puts on its shoes" is often attributed to Mark Twain (although that is contested). Seen in these terms, this short paper amounts to reaching for the shoe rack. In this contribution, I have poked a number of holes in the deglobalisation narrative. No doubt more evidence could be marshalled.

What is particularly jarring about the deglobalisation narrative is that its proponents have not spelt out their desired endgame: what mix of local, regional and global ties they want to see in commerce, what calculus should drive decision-making by business and government, what losses need to be born to reach their nirvana, and what factors might accelerate or impede the transition of the

8 These statistics were obtained from https://hbs.unctad.org/trade-structure-by-partner/.

9 These statistics are taken from this U.S. government source: https://www.census.gov/foreign-trade/Press-Release/edb/edbrel2020.pdf.

10 These statistics were taken from this U.S. government source: https://www.bea.gov/sites/default/files/2021-11/omne1121.pdf. The findings in this paragraph are not materially affected if Saudi Arabia is added to the calculations.

world economy they evidently desire. The deglobalisation narrative is an incomplete prospectus.

From the perspective of international economic governance, other than casting aspersions on the judgement of those that negotiated previous multilateral trade accords and the accession of China to the World Trade Organization, the deglobalisation narrative is silent on how to reform that organisation – or what to salvage from existing global trade rules. Yet rules there will be. Rules on international commerce can be traced back to 1780 BC to the Code of the Babylonian King Hammurabi. Those advancing the deglobalisation narrative should not be allowed to dodge the critical matter of how trading relations between nations with very different economic and political systems should be arranged.

Ultimately, those that proffer the deglobalisation narrative must be held to a higher standard by policymakers, corporate decision-makers, analysts and by anyone giving them a platform to opine on the key building blocks of our standard of living.
